# Efficient *in situ* screening of and data collection from microcrystals in crystallization plates

**DOI:** 10.1107/S2059798324001955

**Published:** 2024-03-15

**Authors:** Amy J. Thompson, Juan Sanchez-Weatherby, Lewis J. Williams, Halina Mikolajek, James Sandy, Jonathan A. R. Worrall, Michael A. Hough

**Affiliations:** a Diamond Light Source Ltd, Harwell Science and Innovation Campus, Didcot OX11 0DE, United Kingdom; b Research Complex at Harwell, Harwell Science and Innovation Campus, Didcot OX11 0DE, United Kingdom; c School of Life Sciences, University of Essex, Wivenhoe Park, Colchester CO4 3SQ, United Kingdom; Stanford University, USA

**Keywords:** serial crystallography, *in situ* data collection, peroxidase, radiation damage, microcrystals

## Abstract

A considerable bottleneck in serial crystallography at XFEL and synchrotron sources is the efficient production of large quantities of homogenous, well diffracting microcrystals. Efficient high-throughput screening of batch-grown microcrystals and the determination of ground-state structures from different conditions is thus of considerable value in the early stages of a project. Here, a highly sample-efficient methodology to measure serial crystallography data from microcrystals by raster scanning within standard *in situ* 96-well crystallization plates is described. Structures were determined from very small quantities of microcrystal suspension and the results were compared with those from other sample-delivery methods. The analysis of a two-dimensional batch crystallization screen using this method is also described as a useful guide for further optimization and the selection of appropriate conditions for scaling up microcrystallization.

## Abstract 2

A sample- and time-efficient method to obtain serial crystallography data from batch-grown microcrystals dispensed as drops on a 96-well crystallization plate is described. This offers a versatile method to obtain low-dose room-temperature structures and guide the optimization of microcrystallization for synchrotron and XFEL serial crystallography experiments.

## 1. Introduction

Serial crystallography using X-ray free-electron lasers (XFELs) and synchrotron beamlines has opened new frontiers for structural biology. Data may be measured from many thousands of microcrystals, enabling very low dose room-temperature structures or, in the case of XFELs, avoiding manifestations of radiation damage due to pulse durations in the tens of femtoseconds (Hough & Owen, 2021 ▸; Horrell *et al.*, 2021 ▸). A key application of serial approaches has been time-resolved crystallography, which uses the ‘pump–probe’ technique. A ‘pump’ is activated to trigger a reaction, with X-ray diffraction data obtained at a defined later time point to ‘probe’ the sample after the reaction has been initiated (Pearson & Mehrabi, 2020 ▸). Common pump triggers that are utilized in structural biology are the mixing of microcrystals with a reagent or the use of a light pulse (Orville, 2020 ▸; Pearson & Mehrabi, 2020 ▸). Other triggers such as temperature or pH jumps have been developed. The time-resolved crystallography field has been described in detail by Pearson & Mehrabi (2020 ▸).

The benefits of room-temperature data collection have been well documented (Thorne, 2023 ▸; Helliwell, 2020 ▸; Fischer, 2021 ▸; Hough *et al.*, 2023 ▸). While the process of protein crystallization always imposes some constraints on protein dynamics due to the requirements of crystal packing and intermolecular interactions, the additional processes of cryoprotection and cooling the crystal to cryogenic temperatures can further constrain or distort the observable conformations derived from the diffraction data. This can result in more potentially biologically relevant conformations being frozen out of the structure compared with the analogous room-temperature structures, which are also significantly closer to physiological temperature. These applications are critically dependent on the availability of large numbers of homogeneous, well diffracting microcrystals. For time-resolved work, fine control of microcrystal size distributions is very helpful for uniform reaction initiation, especially by mixing (Schmidt, 2019 ▸). These techniques are contributing to developments in constructing molecular movies by the interrogation of multiple time points for a deeper understanding of biological mechanisms.

The vast majority of macromolecular crystallization takes place using vapour diffusion in 96-well crystallization plates, which allows effective optimization screening to obtain a desired crystal form. For serial crystallography, however, the most appropriate method is batch crystallization in larger volumes (Stohrer *et al.*, 2021 ▸; Beale *et al.*, 2019 ▸). Commonly, this is conducted in PCR and/or microfuge tubes with volumes of tens of microlitres and the quality of crystals is assessed visually. This is usually performed by using the capability of high-resolution modern optical microscopes to inspect and measure microcrystal size either within the tube or by moving a small quantity of crystals onto a microscope slide. Optimization is conducted by varying the crystallization parameters within tubes to explore the effect of different chemical conditions. The diffraction quality, unit-cell distribution and resolution of any particular batch of crystals is typically not tested regularly during optimization, and promising crystals need to be delivered to the beam using a serial crystallography sample-handling device such as a fixed target (Mueller *et al.*, 2015 ▸), tape drive (Roessler *et al.*, 2013 ▸; Fuller *et al.*, 2017 ▸), gas dynamic virtual nozzle (GDVN; Knoška *et al.*, 2020 ▸), ‘chipless chip’ film sandwich (Doak *et al.*, 2018 ▸) or high-viscosity extruder (Botha *et al.*, 2015 ▸). Preparation for a serial crystallography experiment is also greatly aided by the availability of an initial room-temperature crystal structure determined from the same batch of crystals as used in subsequent experiments. Recent developments have included the use of thin-film sandwiches, where crystals are either grown within or transferred into a sandwich between two layers of polymer film with high X-ray transmission (Axford *et al.*, 2016 ▸). Such a system allows both rotation and serial screening and data collection.

VMXi is a macromolecular crystallography (MX) beamline at Diamond Light Source which currently specializes in measuring data sets *in situ* within 96-well crystallization plates with low X-ray background (Mikolajek *et al.*, 2022 ▸; Sanchez-Weatherby *et al.*, 2019 ▸; Sandy *et al.*, 2024 ▸). Using the VMXi beamline provides an efficient capability for testing the success of vapour-diffusion crystallization experiments and obtaining room-temperature structures. Comparable methods for screening large numbers of crystallization conditions in batch have not yet become available. As VMXi has the capability for both rotation data collection and raster scanning, it is very fast and efficient to set up 2D raster scans over each drop, where a still diffraction image will be taken at each point within the drop, defined in 10 µm step sizes. This is analogous to the chipless chip approach that has been implemented using fixed-target sample stages at XFEL and synchrotron beamlines (Mueller *et al.*, 2015 ▸; Owen *et al.*, 2017 ▸). The automated serial processing pipelines currently implemented at Diamond Light Source (*xia*2.*ssx*; Winter *et al.*, 2022 ▸; Winter, 2010 ▸) can then accept the raster scan as input as though it were a serial experiment performed in a fixed target. Like other serial data-processing software packages, *xia*2.*ssx* is able to process still images with multiple lattices present (Gildea *et al.*, 2014 ▸), which means that overlapping crystals in a position on the raster scan are not problematic. Similarly, the software can handle scenarios in which a particular crystal is exposed more than once during the raster scan. These factors make in-plate screening a highly effective method to quickly screen microcrystals with a high level of automation and has the added benefit of using only very small amounts of sample (<200 nl per drop). This means that with very low sample consumption, insight can be gained as to which crystallization condition is best via diffraction rather than by using visual methods, thus leading to enhanced experimental outcomes. As well as optimized microcrystals, the success of serial crystallography experiments can be further enhanced by testing and optimizing the loading of these crystals into the selected sample-delivery system and the most appropriate choice of beamline parameters and data collection.

For efficient optimization of microcrystallization conditions, a batch-screening diffraction-based technique would be ideal. In this study, we propose that the VMXi beamline provides a straightforward capability for screening batch microcrystallization optimization experiments. We describe a method to rapidly measure serial crystallo­graphy data from aliquots of batch crystallization experiments transferred into a 96-well crystallization plate. This allows either very low volume batches (<10 µl) to be analysed, or a very small fraction of a larger batch to be used while leaving the majority of the sample available for other serial crystallography data collection. Information on crystal quality, unit-cell distribution and any polymorphism within the data can rapidly be established for individual batches and structures can be determined. The resulting structures are comparable with those obtained from the same microcrystals using a conventional sample-delivery approach. We envision that this technique could be adapted to other beamlines with similar *in situ* capabilities where a micro-focused beam and high flux are also used (Broecker *et al.*, 2016 ▸; Axford *et al.*, 2012 ▸), although not all beamlines with *in situ* sample environments use a micro-focused beam with comparable flux to this study; both of these are advisable for routine screening of microcrystals (Bingel-Erlenmeyer *et al.*, 2011 ▸; Okumura *et al.*, 2022 ▸).

## 2. Methods

Batch-prepared microcrystals were dispensed into MiTeGen In Situ-1 plates using a Mosquito liquid dispenser, adding multiple aspiration steps prior to dispensing to ensure that the crystals remained consistently distributed. While samples were dispensed directly from unmodified batch crystallization tubes for this experiment, batch microcrystal suspensions could be concentrated to increase the crystal concentration if required. Where crystals had a tendency to sink to the bottom of the drops during data collection 100 nl drops were used, otherwise 200 nl drops were dispensed. Crystal plates were then stored in a Formulatrix (at 20°C). Crystallization conditions for all samples are provided in the supporting information. Crystal sizes were determined using images taken on the VMXi beamline *in situ*, with the third dimension established from examination of the crystals under a microscope. Raster scans were then performed using the VMXi beamline at 20°C over the selected number of drops using a 10 µm step size (2 × 10^13^ photons per second for 2 ms per image) and a 10 × 10 µm beam of 16.0 keV energy (Sanchez-Weatherby *et al.*, 2019 ▸). The high X-ray energy is used to maximize the resolution and reduce radiation damage (Storm *et al.*, 2021 ▸), and raster scans were performed with no attenuation of the X-ray beam. X-ray diffraction data were recorded using a Dectris EIGER 2X 4M at a distance of 175 mm. These are the standard parameters calibrated for raster scanning at the beamline and are available for users to select through the ISPyB interface (Delagenière *et al.*, 2011 ▸; Fisher *et al.*, 2015 ▸). All still diffraction images were passed into the automated *xia*2.*ssx* pipeline to be processed using *DIALS* as serial data (Winter *et al.*, 2022 ▸; Gildea *et al.*, 2014 ▸). Resolution cutoffs were applied where the CC_1/2_ values fell below 0.3. Refinement was performed in *Phenix* (Liebschner *et al.*, 2019 ▸) against the published room-temperature coordinate files PDB entries 8a9d and 6i43 (Mikolajek *et al.*, 2022 ▸; Ebrahim *et al.*, 2019 ▸), with minor rebuilding such as the modelling of alternative conformations, the removal of side chains without evidence of electron density and the addition/removal of waters as dictated by the electron-density maps. *F*_o_ − *F*_c_ omit maps were generated in *Phenix*. Doses were estimated by *RADDOSE*-3*D* (Bury *et al.*, 2018 ▸).

## 3. Results and discussion

### 3.1. Serial data sets from pre-optimized microcrystals

**Figure 1:**
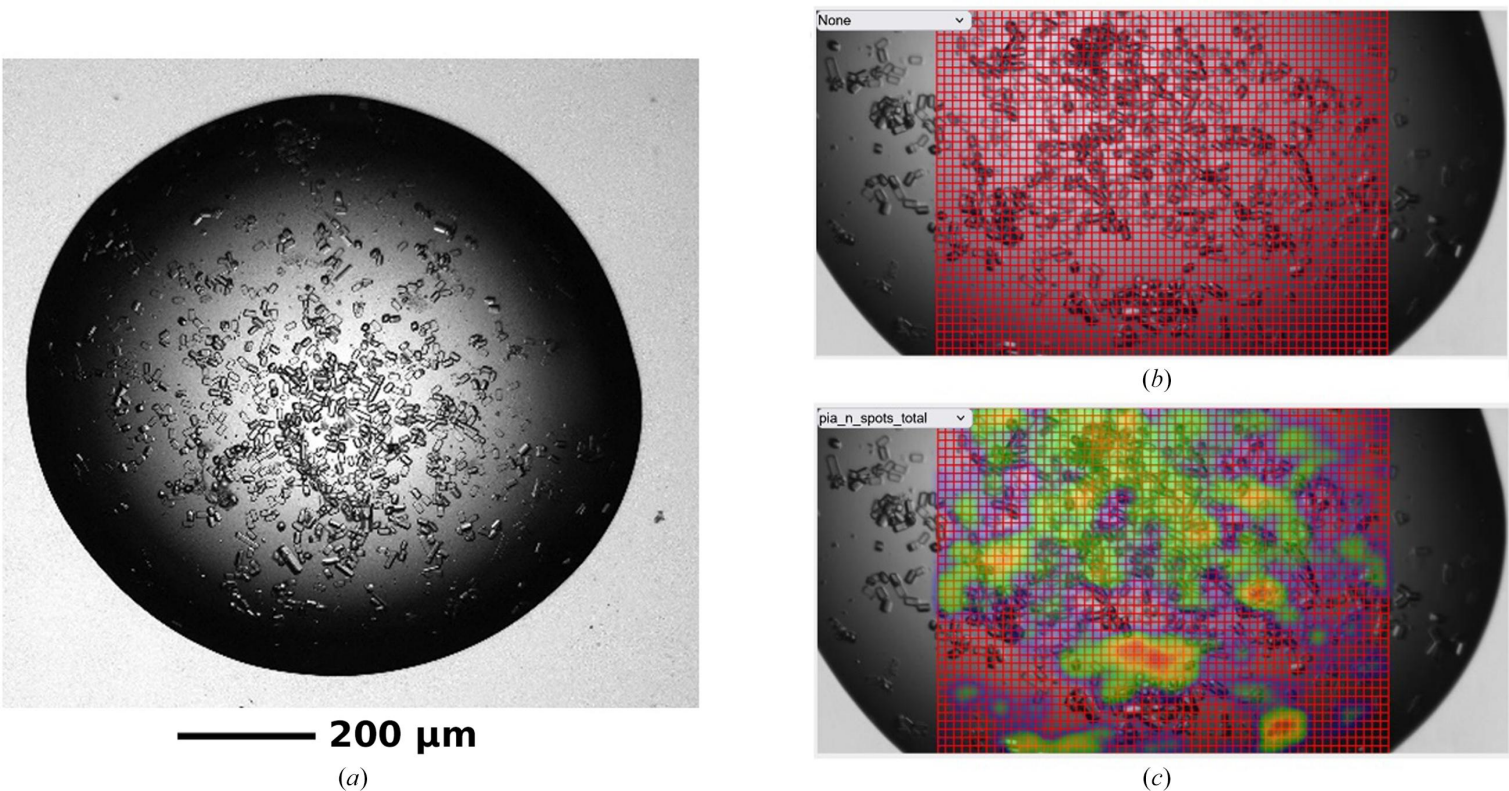
Example raster scan and corresponding heat maps displayed in ISPyB (Delagenière *et al.*, 2011 ▸; Fisher *et al.*, 2015 ▸) as collected on VMXi. (*a*) Visual image of microcrystals of HEWL, (*b*) example raster scan over microcrystals and (*c*) heat map after data collection ranked by the number of spots in each image. Note some small shifts in crystal locations between (*a*) and (*b*)/(*c*). This is a result of the plate being rotated from horizontal to vertical for placement on the beamline goniometer.

**Figure 2:**
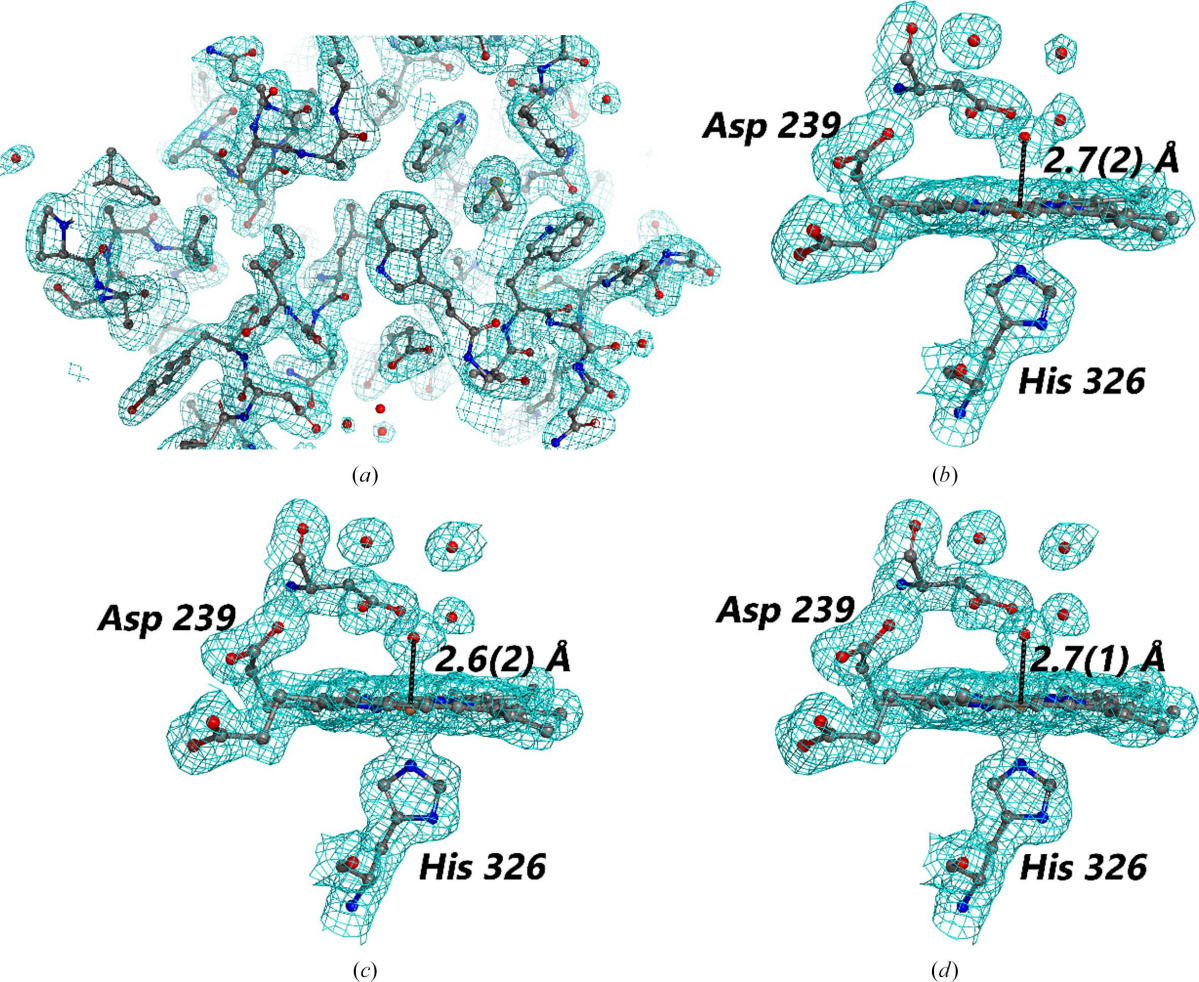
Example 2*F*_o_ − *F*_c_ maps at a contour level of 1σ for (*a*) HEWL, (*b*) DtpAa (eight drops, 5360 merged diffraction patterns), (*c*) DtpAa (12 drops, 10 054 merged diffraction patterns) and (*d*) DtpAa (35 drops, 22 854 merged diffraction patterns). Note that all Fe—O distances are the same within error (shown in parentheses next to the bond length; Kumar *et al.*, 2015 ▸). The structures of DtpAa used previous models as a starting point for refinement (Ebrahim *et al.*, 2019 ▸) and the active site of chain *A* is shown. *F*_o_ − *F*_c_ omit maps showing the presence of clearly defined waters without model bias are included in the supporting information.

As an initial test case for in-plate serial data collection, microcrystals of hen egg-white lysozyme (HEWL; 25 × 10 × 5 µm) were prepared via batch methods (see the supporting information). HEWL was chosen as it is a very well characterized beamline standard and thus is well suited for initial testing of a new method. Raster scans were performed over eight different drops, an example of which is given in Fig. 1 ▸, resulting in a high-quality data set of 9891 merged diffraction patterns (Table 1 ▸). Subsequent refinement against a previously published room-temperature HEWL structure (PDB entry 8a9d; Mikolajek *et al.*, 2022 ▸) and additional rebuilding resulted in a similarly high-quality structure (*R*_work_ = 0.193 and *R*_free_ = 0.231, compared with PDB entry 8a9d, for which *R*_work_ = 0.194 and *R*_free_ = 0.249). However, a higher resolution structure was achieved at 1.88 Å, whereas PDB entry 8a9d only reached 2.10 Å resolution (Table 1 ▸ and Fig. 2 ▸ *a*).

To further develop this technique with a more typical user sample, crystals (30 × 30 × 10 µm) of DtpAa, a heme-containing peroxidase that is a member of the dye-decolourizing peroxidase (DyP) family, were measured in a similar manner (crystal-growth conditions are given in the supporting information). DtpAa crystallized in the monoclinic space group *P*2_1_, representing a much lower symmetry than was the case for HEWL (*P*4_3_2_1_2) and making it an important test system for the general applicability of this method. This protein was chosen as it has well characterized responses to radiation damage, with real-world serial crystallography studies published using both synchrotron and XFEL data (Ebrahim *et al.*, 2019 ▸; Lučić, Chaplin *et al.*, 2020 ▸). The merging statistics for data measured from eight drops of DtpAa microcrystals (5360 merged diffraction patterns) were not as high quality compared with those for HEWL, which is likely to be a consequence of the lower symmetry combined with the greater number of indexed diffraction patterns for HEWL (9891 merged diffraction patterns from eight drops compared with 5360 from DtpAa), although other factors such as the intrinsic crystal quality and subtle differences when interacting with the X-ray beam may also contribute. Data from an additional 27 drops were added to provide a much higher quality data set (increasing the number of merged diffraction patterns to 22 854), although a total of 35 drops is not very efficient for screening if every tested crystallization condition requires 35 drops for low-symmetry space groups. A compromise was found at 12 drops, which provided a higher quality data set than eight drops, but is not significantly worse than 35 drops when considering time and sample efficiency (Table 1 ▸). This is equivalent to half a row of a 96-well (192-drop) crystallization plate and requires approximately one hour on the VMXi beamline (5 min per drop).

The choice of selecting the 12-drop data set as the best when considering quality and efficiency was judged by comparing CC_1/2_ values, *R*_split_ values, completeness, resolution cutoffs, the number of patterns merged and signal-to-noise ratios. While the CC_1/2_ and *R*_split_ values noticeably improved each time on the addition of more data, these changes were approximately linear. However, the increase in completeness from eight to 12 drops was about 2%, while it increased by only 0.02% on increasing from 12 to 35 drops. Additionally, the *I*/σ(*I*) improved significantly from 12.6 to 19.7 when comparing eight and 12 drops, while it only improved marginally from 19.7 to 21.8 when comparing 12 and 35 drops. Using these statistics as an example, it is clear to see that while adding four additional drops to the initial eight-drop data set led to a large increase in data quality (with an additional 20 min of data collection), the addition of 23 extra drops only led to a marginal increase in comparison, but led to a significantly longer data-collection time (nearly two additional hours of data collection). While measurement from 35 drops does constitute a significant time commitment compared with 12 drops, the advantages of sample efficiency cannot be overlooked, and thus the potential time investment and scale-up for low-symmetry space groups should not be a deterrent.

While this is more time-intensive than the 10 min data collections from fixed-target chips (Owen *et al.*, 2017 ▸) and there is less control of dose and higher background from plates, fixed targets require a specific beamline setup, typically use significantly higher sample volumes (50–80 µl per chip versus 1.2 µl for 12 drops in a plate) and some exchange/data collection is not automated. In some cases multiple chips are also required for complete data, which further demonstrates the advantage of the in-plate approach for the purposes of screening and testing. Additionally, other common sample-delivery methods (fixed-target chips, tape drives, GDVNs, ‘chipless chip’ film sandwiches and high-viscosity extruders) generally require upwards of 100 µl of sample for a complete data set (Barends *et al.*, 2022 ▸), making the presented in-plate methodology significantly more sample-efficient.

Subsequent refinement against the previously determined serial femtosecond crystallography (SFX) room-temperature structure (PDB entry 6i43; Ebrahim *et al.*, 2019 ▸) with mutations added (see the supporting information) and additional modelling also yielded high-quality structures and maps (Table 1 ▸). More amino acids, water molecules and alternate conformations could be convincingly modelled when 35 drops were merged (22 854 merged diffraction patterns), although these were removed for the 12-drop and eight-drop data sets (10 054 and 5360 merged diffraction patterns, respectively) where the electron density was not as clear. Despite the difference in merging statistics, the maps for all three were of similar quality, in particular noting the well defined waters around the heme in chain *A* in each case (Fig. 2 ▸). The electron density corresponding to the Fe—OH_2_ bond is also marginally clearer in the maps from 12 and 35 wells (10 054 and 22 854 merged diffraction patterns, respectively), but this would not hinder map interpretation from the eight-drop data set (5360 merged diffraction patterns) within a screening context. Good map quality has been shown to still occur in serial crystallo­graphy data even when the merging statistics are poor, so these results are not unexpected (Moreno-Chicano *et al.*, 2019 ▸). These two examples demonstrate the capacity for high-quality serial structures to be obtained by performing raster scans *in situ* in crystallization trays using a minimum quantity of material and experimental time.

We have previously described dose-dependent structures of DtpAa obtained using a fixed target (Ebrahim *et al.*, 2019 ▸), and a comparison of data-quality indicators is given in the supporting information. The resolution achieved in the fixed-target experiment was 1.78 Å (8596 merged diffraction patterns; Ebrahim *et al.*, 2019 ▸), while the in-plate data from 35 drops reached 1.79 Å resolution (22 854 merged diffraction patterns). The in-plate data from 12 drops (10 054 merged diffraction patterns) are most comparable in terms of number of hits, and the resolution reached 1.88 Å, which is only marginally lower than the fixed-target data set. The crystals for the fixed-target data set were marginally smaller, measuring 15 × 15 µm, with a beam of comparable size and approximately an order of magnitude less flux (Sanchez-Weatherby *et al.*, 2019 ▸; Ebrahim *et al.*, 2019 ▸). Comparable CC_1/2_ and *R*_split_ values were observed, although direct comparison is difficult due to variations in the number of merged diffraction patterns. In previous structures free of radiation-induced changes (measured with 10 fs XFEL pulses) the Fe—OH_2_ bond length has consistently been shown to be ∼2.4 Å and to progressively increase in a quasi-linear manner with accumulated dose until the water molecule is at a distance that indicates complete dissociation from the heme subsequent to reduction of iron from the ferric to the ferrous state (Ebrahim *et al.*, 2019 ▸). The first serial synchrotron crystallography (SSX) structure in that study showed this bond at 2.48 Å in comparison to the SFX value of 2.4 Å. The observed Fe—O distances of 2.6–2.7 Å in the structures presented here are similar to those observed at doses of 75–100 kGy in experiments where a sequence of still diffraction patterns were obtained from each microcrystal in a fixed target over several tens of milliseconds, providing a fine-grained dose series. The dose delivered in this data collection from crystallization plates was approximately 18 kGy per crystal (and hence for the full data set as each crystal is assumed to be exposed only once), which is significantly lower than would be expected for the observed Fe—O bond length when compared with the SSX data. The explanation for this apparent discrepancy may come from the nature of measurements within the crystallization drop, where free radicals and electrons generated by the X-ray beam are able to migrate (Pfanzagl *et al.*, 2020 ▸; Kekilli *et al.*, 2017 ▸; Beitlich *et al.*, 2007 ▸). While at 100 K a range of 4 µm for photoelectrons has been demonstrated (leading to a suggested 17 µm translation of a 10 µm beam to avoid re-exposing damaged regions of the crystal), the range of mobile free radicals and electrons at room temperature is likely to be larger (Sanishvili *et al.*, 2011 ▸). One room-temperature radiation-damage study used a 10 µm separation between exposures with a 3 × 1.5 µm beam in order to avoid re-exposing damaged regions (de la Mora *et al.*, 2020 ▸). This leads to an X-ray dose that is less controlled and which may be underestimated in comparison to that from a fixed-target experiment, where crystals are isolated from each other and the dose per crystal is identical.

It is prudent to compare the outcomes of our *in situ* approach with previous data obtained using fixed-target and high-viscosity extruder sample-delivery methods at synchrotron and XFEL sources. The resolution is comparable with that obtained previously, although of course this depends on the number of crystals merged, the source and the beamline. For a particular batch of crystals the resolution achieved *in situ* would be expected to be slightly lower than in, for example, a fixed target or thin-film sandwich due to the increased background of the crystallization plate (Axford *et al.*, 2016 ▸), but may be considered to be comparable to an extruder experiment where there is considerable background from the crystal-carrying medium. Note that we have used a MiTeGen In Situ-1 plate, where the base of the well is made of 100 µm thick COC film to enable measurements from small crystals with very low background from the plate (Soliman *et al.*, 2011 ▸). This must be balanced against the ease of use and utility of measuring data and obtaining initial structures straightforwardly within a crystallization plate.

### 3.2. Serial data sets using crystals from a microcrystallization screening experiment

**Figure 3:**
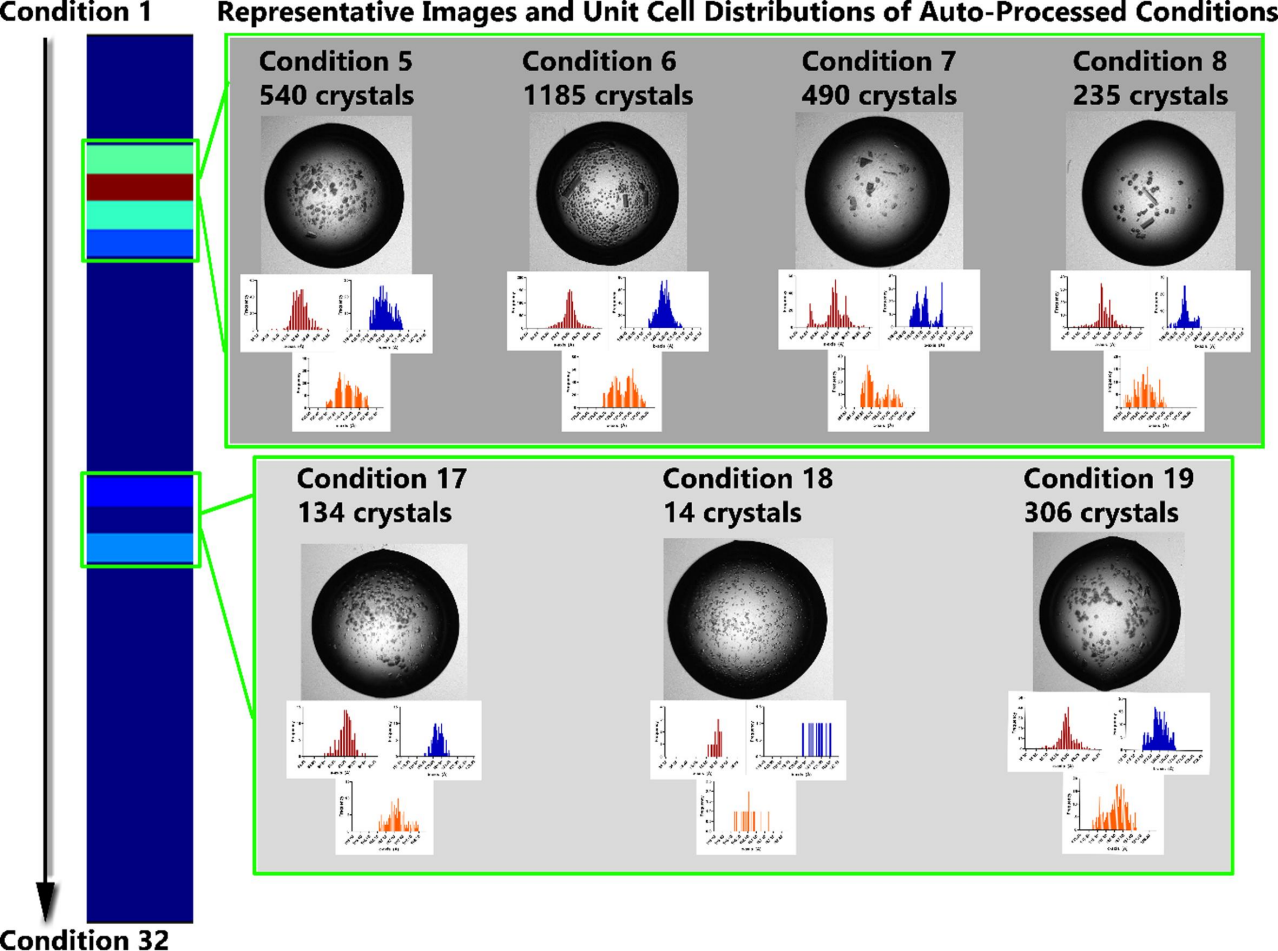
Outcomes of the DtpB crystallization screen. The blue bar on the left is a heat map that corresponds to data sets which could be automatically processed through *xia*2*.ssx*, with colour corresponding to the number of merged crystals per condition (dark blue = 0, red = 1185 merged diffraction patterns). For each identified hit, the corresponding crystal images are provided, along with unit-cell distributions (*a* axis, red; *b* axis, blue; *c* axis, orange). The unit-cell distributions are included for visualization; enlarged versions are included in Section S3.3 for full readability.

To demonstrate the capability to screen microcrystallization conditions using this technique, a batch crystallization optimization screen of a second DyP heme enzyme, DtpB (Lučić, Svistunenko *et al.*, 2020 ▸; Lučić *et al.*, 2022 ▸), was dispensed into crystallization trays for serial data collection. DtpB was chosen for this test case due to its similarity to DtpAa as well as the availability of a recently prepared batch crystallization screen. While adequate data were shown for monoclinic crystals of DtpAa with only 12 drops merged (10 054 merged diffraction patterns), this also represented a pre-optimized test case. Here, we define adequate data to be complete, with a CC_1/2_ of greater than 90%, and composed of over 5000 diffraction patterns (Mehrabi *et al.*, 2021 ▸). As a real screen is likely to have lower crystal quality and concentration, each test condition for DtpB was dispensed into an entire row of the crystallization plate (12 wells with two drops each). The protein concentration was kept consistent and a range of HEPES, PEG and MgCl_2_·6H_2_O concentrations in the precipitant solution were tested. The average molecular weight of PEG was also varied as well as the pH and the protein stock to mother liquor ratio (see the supporting information for the exact conditions). This amounted to 32 conditions spread over four crystallization plates, each containing eight different crystallization conditions dispensed over a row (100 nl per drop). The plates were then imaged, and each drop was measured with a raster scan on VMXi using the same beamline parameters. Although all 32 tested crystallization conditions were passed to the *xia*2.*ssx* pipeline, where drops with identical crystallization conditions were combined, only seven could successfully be processed automatically. These seven hits came from two groups of similar crystallization conditions (Fig. 3 ▸).

Both groups of crystal hits had the same mother-liquor compositions but varied in the ratio of mother liquor to protein stock. MgCl_2_·6H_2_O and HEPES each at a concentration of 125 m*M* resulted in crystals, with variations within each group of hits being driven by varying PEG concentrations. The first group of hits comprised a 1:1 ratio of mother liquor and protein stock, while the second group of hits had a 2:1 ratio. While a comparison of merging statistics is useful, microscopic examination of the crystallization drops was performed first to provide an extra level of filtering. As seen for both groups of conditions, the majority of the ‘hits’ also contained a large amount of noncrystalline material in the drops (Fig. 3 ▸). Inspection of the diffraction heat maps supported this conclusion (see the supporting information). This is not unexpected, as they are chemically close to optimized crystallization conditions, but the subtle differences do not result in perfect crystal growth. Using samples with a significant concentration of noncrystalline material or side product in any kind of soaking experiments can cause unexpected reactions or may even block an extruder delivery system. Therefore, only the two hits which were able to be automatically merged and confirmed to be fully crystalline were considered further (condition 7 and condition 18 in Fig. 3 ▸). While this currently requires an element of manual inspection and analysis, it is envisioned that future developments to improve the current image-recognition algorithms (Mikolajek *et al.*, 2023 ▸) and to develop automatic analysis of raster-scan diffraction data could extract information such as crystal size, type (protein, salt *etc.*) and distribution.

Both fully crystalline hits occurred when the mother liquor contained 18% PEG 4000, and they only differed in the ratio of mother liquor to protein stock. The first hit (1:1 protein:mother liquor, condition 7) had considerably more crystals that were of sufficient quality for auto-processing compared with the second hit (2:1 protein:mother liquor, condition 18), although the unit-cell distributions for the first hit are far from ideal. While it is difficult to judge the quality based on unit-cell distribution for the second hit (due to the very low hit rate), the lack of a Gaussian distribution for the first hit is notable. Additionally, the second hit visually appears to contain crystals that are more homogenous in size, and it is possible that the low hit rate is due to the significantly smaller size of the crystals (13 × 8 µm) compared with the first hit (50 × 30 µm), where background scatter can begin to interfere with the ability to distinguish weak diffraction. Other factors which may contribute to fewer identifiable hits are the inherent sample quality, which is not expected to be as high as possible from an initial crystallization screen. While the exact reason has not been determined here, as the purpose of this experiment was to demonstrate how this technique can direct a serial crystallography project, a diffracting sample could be quickly found and feedback provided to direct the next course of action for the user.

### 3.3. Ease of use and limitations

In the work presented here, microcrystals were grown in batch and small volumes were transferred to a standard *in situ* crystallization plate for data collection. This allows crystallization-condition space to be explored across a range of batch crystallization trials, but also allows room-temperature serial crystallographic structures to be determined straightforwardly during the normal operation of VMXi in plate mode. Structures can easily be determined from very small quantities of microcrystal suspension, equating to very small masses of protein (only 3 µg for 10 054 merged diffraction patterns of DtpAa). Using this method, static structure determination of ground states and stable reaction intermediates or ligand-bound forms are straightforward. For these types of experiment, where there is no time-dependence or requirement for crystal homogeneity, in-plate data collection may well be sufficient to answer the particular scientific question.

We propose that this capability is potentially of great use during the earlier stages of serial crystallography projects to obtain initial structures and optimize microcrystal production. While initial, unoptimized screens may not produce crystals of sufficient size/quality for full structure refinement at this stage, they provide time-saving direction for further optimization, the outcomes of which can then be quickly measured using these methods if an initial room-temperature structure is required. In this approach, rather than optimizing crystallization conditions from several different starting points or ‘hits’, the most promising hits from each round of optimization can quickly be identified and used as the basis of future iteration.

Users do not need to wait for a dedicated serial crystallo­graphy experiment to obtain initial information about their microcrystal system and structure. Indeed, for several applications such as the addition of different ligands to batch microcrystals, the in-plate approach provides a straightforward and rapid method of data collection. Projects could then transfer to more conventional sample-delivery methods, for example time-resolved experiments using sample-delivery systems such as fixed targets or droplet-on-demand tape-drive systems, once the crystallization conditions have reliably been optimized and an initial room-temperature structure has been obtained (Barends *et al.*, 2022 ▸). The requirement to shoot samples in plates does limit the size of the crystals that can be reliably measured due to the increased background scatter affecting data processing, but as this study shows, even if small, low-quality crystals cannot provide a refined structure from an initial screen, they can still be identified as a hit. With this study providing a successful proof-of-concept for testing microcrystals, further developments on VMXi are now planned to increase automation and progress towards thin-film screening to reduce background and improve the signal to noise for weakly diffracting crystals (Axford *et al.*, 2016 ▸).

Because the crystals are within aqueous droplets, the potential exists for X-ray-induced chemistry to spread between the X-ray exposures either because the microcrystal is larger than the X-ray beam or by the diffusion of X-ray-generated radicals and electrons through the droplet as the raster scan proceeds. In the peroxidase sample described here, the length of a particular bond acts as a molecular ruler of X-ray-induced photoreduction. The doses calculated for in-plate data collection appeared to be lower than suggested by the electron density, although it is important to note that time regimes and sample environments differ from those in the previous fixed-target experiment. It is likely that for the raster-scanning serial approach the propagation of X-ray-generated electrons and free radicals and the potential multiple exposure of large crystals leads to an underestimate of the dose. However, for the cases and applications that we describe this is not a significant limitation.

Interested users can find information about accessing VMXi beamtime on the Diamond Light Source website (https://www.diamond.ac.uk/Instruments/Mx/VMXi.html) and in recent publications (Mikolajek *et al.*, 2022 ▸; Sandy *et al.*, 2024 ▸).

## 4. Conclusions

We demonstrate that raster-scan serial data collection within crystallization plates can be an easily accessible and rapid method of assessing microcrystallization experiments and determining suitable-quality room-temperature structures. Such a method is highly complementary to existing serial synchrotron and XFEL data-collection methods and is particularly applicable at the earliest stages of a project. In-plate data collection is very sample-efficient and time-efficient. The data obtained can allow a more rapid progression of projects towards time-resolved experiments and help to make more effective use of scarce XFEL beamtime. Implementation of user-friendly, routine thin-film screening on VMXi to decrease the background is currently in development to improve issues with background from plate-based sample handling, as well as the development of more sophisticated algorithms to analyse raster-scan data to provide more in-depth feedback to users.

## 5. Related literature

The following reference is cited in the supporting information for this article: Lučić *et al.* (2023 ▸).

## Supplementary Material

PDB reference: HEWL, 8rge

PDB reference: DtpAa, 8 drops, 8rgy

PDB reference: DtpAa, 12 drops, 8rgw

PDB reference: DtpAa, 35 drops, 8rgs

Supporting information including Supplmentary Tables and Figures. DOI: 10.1107/S2059798324001955/wa5148sup1.pdf

## Figures and Tables

**Table 1:** Data-collection and refinement statistics from in-plate serial experiments Values for the highest resolution shell are shown in parentheses. The volume of batch-grown microcrystal suspension dispensed into each drop is indicated. All data were measured at a wavelength of 0.775 Å.

	HEWL (8 drops, PDB entry 8rge)	DtpAa (8 drops, PDB entry 8rgs)	DtpAa (12 drops, PDB entry 8rgw)	DtpAa (35 drops, PDB entry 8rgy)
Diffraction-weighted dose (kGy)	33	18	18	18
Resolution range (Å)	55.56–1.88 (1.95–1.88)	69.76–2.07 (2.14–2.07)	69.73–1.88 (1.95–1.88)	69.77–1.79 (1.85–1.79)
Space group	*P*4_3_2_1_2	*P*2_1_	*P*2_1_	*P*2_1_
*a*, *b*, *c* (Å)	78.57, 78.57, 37.77	72.46, 67.76, 74.71	72.42, 67.73, 74.67	72.47, 67.77, 74.73
α, β, γ (°)	90, 90, 90	90, 105.69, 90	90, 105.69, 90	90, 105.70, 90
No. of diffraction patterns merged	9891	5360	10054	22854
Volume dispensed	1.6 µl [200 nl per drop]	0.8 µl [100 nl per drop]	1.2 µl [100 nl per drop]	3.5 µl [100 nl per drop]
Total reflections	1117460 (32281)	1220378 (47508)	2721999 (56731)	6511950 (151249)
Unique reflections	10076 (973)	42538 (4203)	56729 (5631)	65821 (6543)
Multiplicity	110.9 (65.1)	28.7 (22.9)	48.0 (25.7)	98.9 (45.8)
Completeness (%)	99.27 (99.90)	97.74 (84.49)	99.73 (99.36)	99.75 (99.69)
Mean *I*/σ(*I*)	19.1 (1.2)	12.6 (2.0)	19.7 (2.2)	21.8 (2.0)
Wilson *B* factor (Å^2^)	31.36	25.18	20.43	22.00
*R*_split_	0.083 (1.143)	0.285 (1.759)	0.203 (1.061)	0.121 (0.978)
CC_1/2_	0.997 (0.398)	0.927 (0.329)	0.958 (0.302)	0.990 (0.315)
Reflections used in refinement	10002 (972)	41631 (3553)	56581 (5597)	65657 (6542)
*R*_work_	0.1925 (0.2832)	0.2233 (0.3855)	0.2115 (0.2927)	0.1885 (0.3265)
*R*_free_	0.2305 (0.4288)	0.2748 (0.4277)	0.2420 (0.3491)	0.2281 (0.3893)
Water molecules	83	412	412	417
Protein residues	129	724	724	728
R.m.s.d., bond lengths (Å)	0.007	0.003	0.003	0.009
R.m.s.d., angles (°)	0.90	0.62	0.66	1.05
Ramachandran favoured (%)	98.43	98.33	98.33	98.47
Ramachandran allowed (%)	1.57	1.67	1.67	1.53
Ramachandran outliers (%)	0.00	0.00	0.00	0.00
Rotamer outliers (%)	1.72	0.74	0.37	0.55
Clashscore	4.19	2.55	2.37	2.26
Average *B* factors (Å^2^)				
Overall	34.64	27.62	21.46	23.16
Macromolecules	33.87	27.36	21.13	22.80
Solvent	44.69	32.87	27.42	29.41
