# Supplementary material for: Efficient in situ screening of and data collection from microcrystals in crystallization plates
Source: Acta Crystallogr D Struct Biol. 2024 Mar 15;80(Pt 4):279–88. doi: 10.1107/S2059798324001955 (PMC10994175; doi:10.1107/S2059798324001955)
Supplement: Supplementary file 1 [file d-80-00279-sup1.pdf]

STRUCTURAL BIOLOGY

Volume 80 (2024)

Supporting information for article:

Efficient *in situ* screening of and data collection from microcrystals in crystallization plates

Amy J. Thompson, Juan Sanchez-Weatherby, Lewis J. Williams, Halina Mikolajek, James Sandy, Jonathan A. R. Worrall and Michael A. Hough

S1. Grid Scanning Methodology

Rectangular grids were placed over the drop with the aim of maximising the area covered without extending beyond the drop. While this does not cover all regions of the drop, this was done to maximise data collection and processing efficiency, as covering the entire drop would result in numerous blank frames. Current software limitations require all grids to be rectangular. It is also worth noting that the drops are not always centred correctly by the Mosquito dispensing system, meaning that sometimes parts of the drop are not visible on the images, and thus not able to be shot on the beamline.

S2. Lysozyme (HEWL)

S2.1. Crystallisation Conditions

HEWL was diluted in water to a concentration of 50 mg mL^{-1} and mixed in a 1:1 ratio with crystallisation buffer (1M acetic acid, 5% PEG 6000, 20% NaCl, pH 3.0). The pH of the buffer was adjusted with NaOH and acetic acid. The crystals were vortexed at 22 °C and then left for 30 minutes in a temperature block (also at 22 °C). Crystals of dimensions 25 x 10 µm were stored at 4 °C. before transfer into crystallisation plates which were then incubated in the beamline crystal hotel at 20° C.

S3. DtpAa

S3.1. Crystallisation Conditions

The Y389F variant of DtpAa was used in this study (Lucic *et al.*, 2023). The purified DtpAa variant was subjected to batch of micro-crystallisation in 1.5 ml Eppendorf tubes using a 1:3 v/v ratio of 10 mg/ml protein in 20 mM sodium phosphate, 150 mM NaCl, pH 7.0 and a mother liquor solution containing 12% v/v PEG 3350, 100 mM HEPES pH 7.0 to give a final solution volume of 400 µl. Batches were set up within 48 h of protein purification. Crystals of dimensions 30 x 30 µm appeared over a 24-48 h period with incubation at 18°C. Following transfer to crystallisation plates crystals were stored in a sample hotel at the beamline 20° C prior to data collection.

S3.2. OMIT Maps for Water Position

During the refinement process, to determine if the four water atoms around the heme were truly present or just appearing in the electron density due to model bias, torsion angle simulated annealing OMIT maps were also generated in Phenix with the water molecules omitted in refinement. As more crystals from more drops were merged together, more of the heme pocket waters could be unambiguously identified, with all four showing clear density in the Fo-Fc maps once 35 drops (22,854 merged diffraction patterns) were merged (Figure S1).

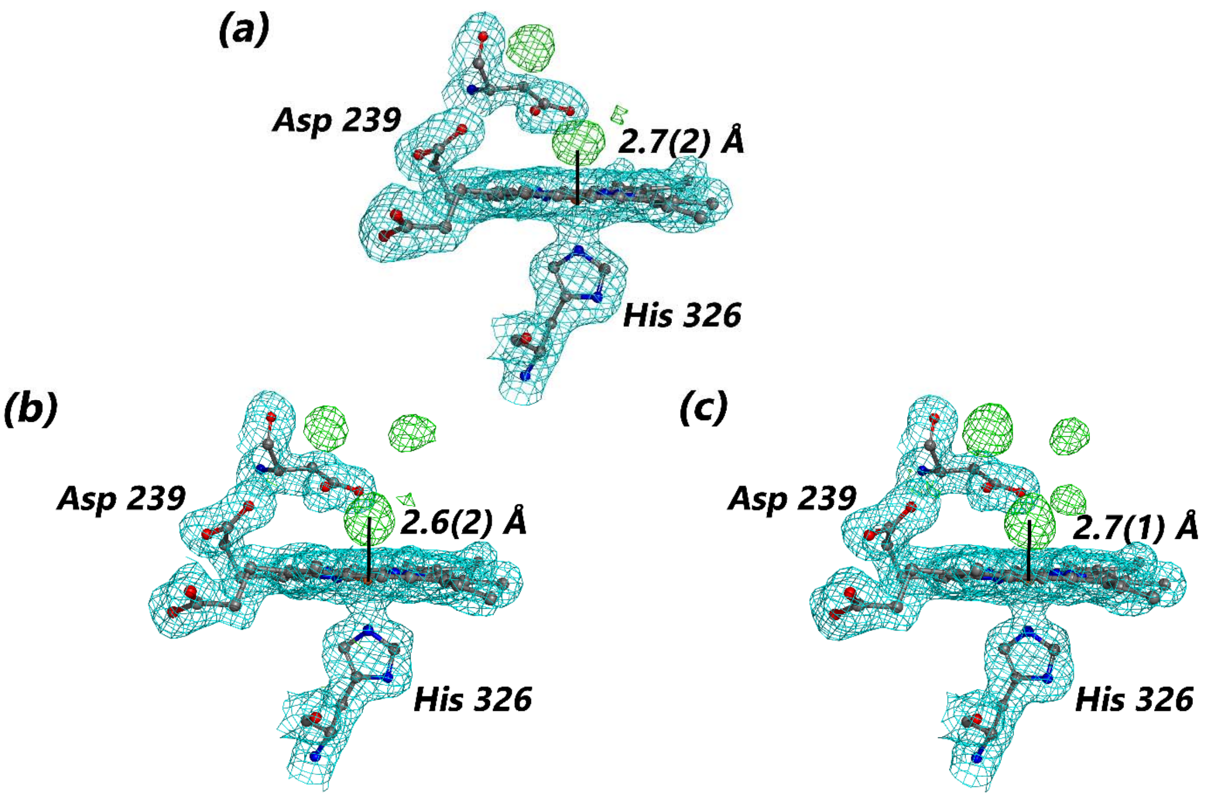

Figure S1 Simulated annealing OMIT maps were calculated with the four waters around the Heme active site removed for DtpAa. Fo-Fc maps shown in green (contour level 3), while 2Fo-Fc maps are shown in blue (contour level 1). When there are only 8 drops (5,360 diffraction patterns) merged (a), only two waters are unambiguously present when model bias is removed. This increases to three waters when 12 drops (10,054 diffraction patterns) are merged together (b), and all four waters can be identified in the OMIT maps when 35 drops (22,854 diffraction patterns) are merged.

S3.3. Comparison to Previously Published Fixed-Target Data

We have previously determined the structure of ferric DtpAa by SFX at SACLA using microcrystals (15 x 15 µm) in fixed targets to 1.88 Å resolution (PDB 6I43). Superposition of the VMXi structures with this gave all atom RMSD values for chain A of 0.111 Å (36 drop, 22,854 merged diffraction patterns), 0.126 Å (12 drop, 10,054 merged diffraction patterns) and 0.127 Å (8 drop, 5,360 merged diffraction patterns) (see Figure S2 for visualisation). Comparable SSX structures are that from a low dose fixed target experiment at I24, Diamond (PDB 6I7Z and 6I8O) to 1.78 Å resolution (crystals also 15 x 15 µm). (Ebrahim *et al.*, 2019). Comparison of data quality statistics are in Table S1 demonstrating that all structures are comparable in terms of quality and structure.

Table S1 In-plate serial structures of DtpAa compared to fixed target structures obtained at Diamond I24 and SACLA. All structures are in space group P21.

	DtpAa (8 drops) 8GRS	DtpAa (12 drops) 8RGW	DtpAa (35 drops) 8RGY	DtpAa SSX 6I7Z	DtpAa SSX 6I8O	DtpAa SACLA 6I43
Wavelength (Å)	0.775	0.775	0.775	0.9686	0.9686	1.24
Absorbed dose (kGy)	18	18	18	32.8	39.2	N/A
Resolution range (Å)	69.76 – 2.07 (2.144 – 2.07)	69.73 – 1.88 (1.947 – 1.88)	69.77 – 1.79 (1.854 – 1.79)	44.64 - 1.78 (1.81 - 1.78)	44.71 – 1.70 (1.73 – 1.70)	41.46 - 1.88 (1.91 - 1.88)
Unit cell (Å,°)	72.46 67.76 74.71 90 105.69 90	72.42 67.73 74.67 90 105.69 90	72.47 67.77 74.73 90 105.70 90	72.95 68.30 74.78 90 105.68 90	72.99 68.36 74.95 90 105.63 90	72.72 68.18 74.62 90 105.58 90
No. diffraction patterns merged	5,360	10,054	22,854	9,751	14,719	73,281
No. drops or chips	8	12	35	1	3	11
Volume dispensed	0.8 µL (100 nL/drop)	1.2 µL (100 nL/drop)	3.5 µL (100 nL/drop)	100 – 200 µL per chip	100 – 200 µL per chip	100 – 200 µL per chip
Unique reflections	42,538 (4,203)	56,729 (5,631)	65,821 (6,543)	67,891	78,148	57,312
Multiplicity	28.7 (22.9)	48.0 (25.7)	98.9 (45.8)	27.12 (11.36)	52.99 (12.44)	380.6 (212.6)
Completeness (%)	97.74 (84.49)	99.73 (99.36)	99.75 (99.69)	100 (99.9)	100 (99.9)	100 (100)

Mean I/sigma (I)	12.6 (2.0)	19.7 (2.2)	21.8 (2.0)	3.1	2.31 (0.58)	9.6 (1.73)
Wilson B-factor (Å^2)	25.18	20.43	22.00	N/A	N/A	N/A
R-split	0.285 (1.759)	0.203 (1.061)	0.121 (0.978)	0.189 (0.4772)	0.182 (0.7381)	0.0722 (0.5829)
$CC_{1/2}$	0.927 (0.329)	0.958 (0.302)	0.990 (0.315)	0.944 (0.677)	0.959 (0.500)	0.993 (0.722)
Reflections used in refinement	41,631 (3,553)	56,581 (5,597)	65,657 (6,5423)	67,891	78,148	57,312
R-work	0.2233 (0.3855)	0.2115 (0.2927)	0.1885 (0.3265)	0.1647	0.1805	0.1320
R-free	0.2748 (0.4277)	0.2420 (0.3491)	0.2281 (0.3893)	0.2032	0.2337	0.1670
Water molecules	412	412	417	362	491	481
Protein Residues	724	724	728	724	724	724
RMS(bonds, Å)	0.003	0.003	0.009	0.006	0.007	0.009
RMS(angles, °)	0.62	0.66	1.05	0.81	0.85	0.92
Ramachandran favoured (%)	98.33	98.33	98.47	98.3	97.91	98.5
Ramachandran allowed (%)	1.67	1.67	1.53	1.67	2.09	1.50
Ramachandran outliers (%)	0.00	0.00	0.00	0.00	0.00	0.00
Rotamer outliers (%)	0.74	0.37	0.55	0.00	0.00	0.75
Clashscore	2.55	2.37	2.26	3.42	4.04	0.28
Average B-factor (Å^2)	27.62	21.46	23.16	22.6	23.9	34.6

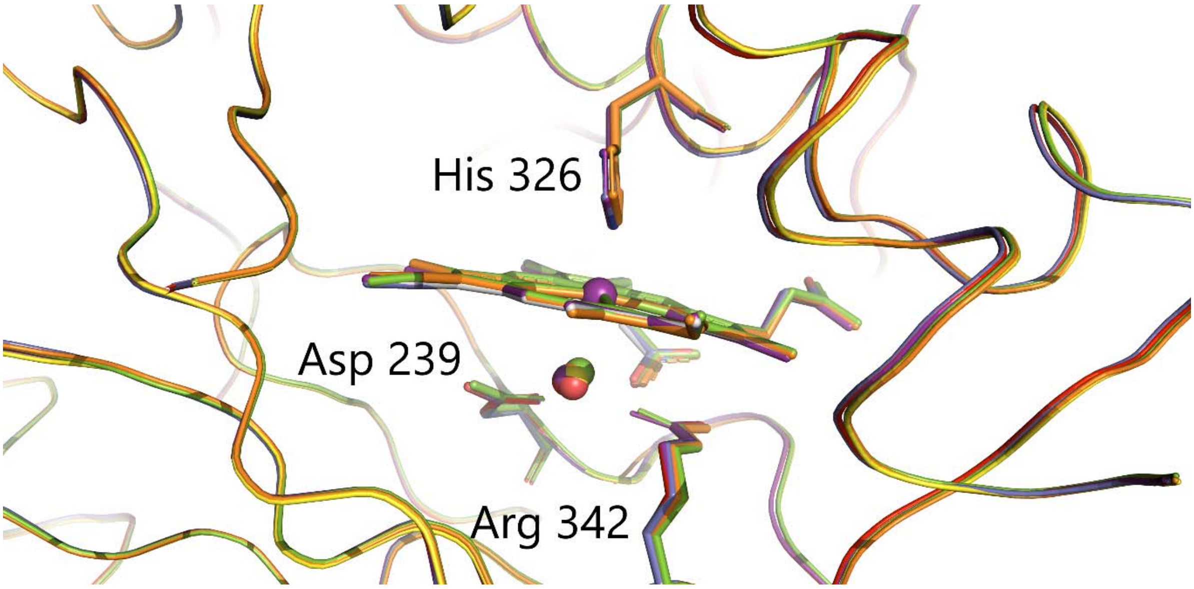

Figure S2 Superposition of DtpAa (8 drops, 5,360 crystals), DtpAa (12 drops, 10,054 crystals), DtpAa (35 drops, 22854 crystals), 6I7Z, 6I43 and 6I8O (colours in order: blue, purple, red, orange, green, yellow). The active site of Chain A is shown. The heme Fe and coordinated water molecules are shown as spheres, while remaining heme atoms and key active site residues are shown as sticks.

S4. DtpB

S4.1. Crystallisation Screen Conditions

For DtpB screening, all microcrystal batches were set up in 1.5 ml Eppendorf tubes with a total solution volume of 200 µl. The protein stock used in all conditions was 6.2 mg/ml of DtpB in 20 mM sodium phosphate, 150 mM NaCl, pH 7.0. All batches were grown at 18°C (Table S2).

Table S2 DtpB Screening Conditions

Condition	Mother Liquor	Protein:Mother Liquor Ratio
1	50 mM $MgCl_2(H_2O)_6$, 100 mM HEPES pH 7.5, 30% PEG MME 550	1:1
2	100 mM HEPES pH 7.0, 10% PEG 3350	1:1
3	100 mM HEPES pH 7.0, 15% PEG 3350	1:1
4	100 mM HEPES pH 7.0, 12% PEG 4000	1:1
5	125 mM $MgCl_2(H_2O)_6$, 125 mM HEPES pH 7.5, 12% PEG 4000	1:1
6	125 mM $MgCl_2(H_2O)_6$, 125 mM HEPES pH 7.5, 15% PEG 4000	1:1
7	125 mM $MgCl_2(H_2O)_6$, 125 mM HEPES pH 7.5, 18% PEG 4000	1:1
8	125 mM $MgCl_2(H_2O)_6$, 125 mM HEPES pH 7.5, 20% PEG 4000	1:1

9	100 mM HEPES pH 7.0, 10% PEG 6000	1:1
10	100 mM HEPES pH 7.0, 17% PEG 6000	1:1
11	100 mM HEPES pH 7.0, 20% PEG 6000	1:1
12	50 mM $MgCl_2(H_2O)_6$, 100 mM HEPES pH 7.5, 30% PEG MME 550	1:2
13	100 mM HEPES pH 7.0, 10% PEG 3350	1:2
14	100 mM HEPES pH 7.0, 15% PEG 3350	1:2
15	100 mM HEPES pH 7.0, 12% PEG 4000	1:2
16	125 mM $MgCl_2(H_2O)_6$, 125 mM HEPES pH 7.5, 12% PEG 4000	1:2
17	125 mM $MgCl_2(H_2O)_6$, 125 mM HEPES pH 7.5, 15% PEG 4000	1:2
18	125 mM $MgCl_2(H_2O)_6$, 125 mM HEPES pH 7.5, 18% PEG 4000	1:2
19	125 mM $MgCl_2(H_2O)_6$, 125 mM HEPES pH 7.5, 20% PEG 4000	1:2
20	100 mM HEPES pH 7.0, 10% PEG 6000	1:2
21	100 mM HEPES pH 7.0, 17% PEG 6000	1:2
22	100 mM HEPES pH 7.0, 20% PEG 6000	1:2
23	50 mM $MgCl_2(H_2O)_6$, 100 mM HEPES pH 7.5, 30% PEG MME 550	1:3
24	100 mM HEPES pH 7.0, 10% PEG 3350	1:3
25	100 mM HEPES pH 7.0, 15% PEG 3350	1:3
26	100 mM HEPES pH 7.0, 12% PEG 4000	1:3
27	125 mM $MgCl_2(H_2O)_6$, 125 mM HEPES pH 7.5, 12% PEG 4000	1:3
28	125 mM $MgCl_2(H_2O)_6$, 125 mM HEPES pH 7.5, 15% PEG 4000	1:3
29	125 mM $MgCl_2(H_2O)_6$, 125 mM HEPES pH 7.5, 18% PEG 4000	1:3
30	125 mM $MgCl_2(H_2O)_6$, 125 mM HEPES pH 7.5, 20% PEG 4000	1:3
31	100 mM HEPES pH 7.0, 10% PEG 6000	1:3
32	100 mM HEPES pH 7.0, 17% PEG 6000	1:3

S4.2. Example Heat Map of DtpB

For cases where non-crystalline material was visually identified, the heat maps from the grid scans can be examined to determine crystallinity based on diffraction. An example is given in Figure S3 where condition 6 was identified as a crystallographic 'hit' but was visually non-homogeneous with what appears to be a mixture of crystalline and non-crystalline material. The heat map corresponds to the number of diffraction spots in each position of the grid and demonstrates that a significant portion

of the material in the drop does not diffract. Therefore, even where good statistics and unit cell distributions may arise, the ability to quickly scan through these statistics mapped to a diffraction heat map is useful for rapid screening of microcrystals for overall batch sample quality, not just crystal quality.

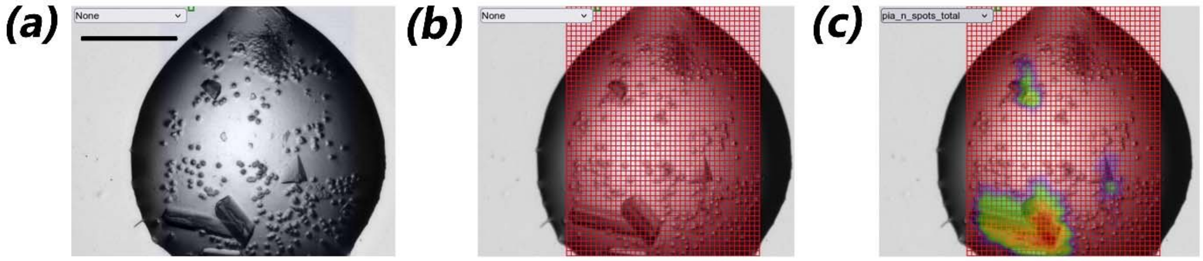

Figure S3 An example of using diffraction to verify the distinction between crystalline and non-crystalline material (condition 6). (a) The beamline in-focus image prior to data collection, (b) the grid scan that was performed overlaid on top of the beamline in-focus image, (c) heat-map applied to the grid scaled by number of diffraction spots identified at each position. The black bar in (a) is 200 µm for scale.

S4.3. Unit Cell Distributions of Identified Hits

Enlarged versions of the unit cell distributions in Figure 3 are given in this section (Figure S4 – S10).

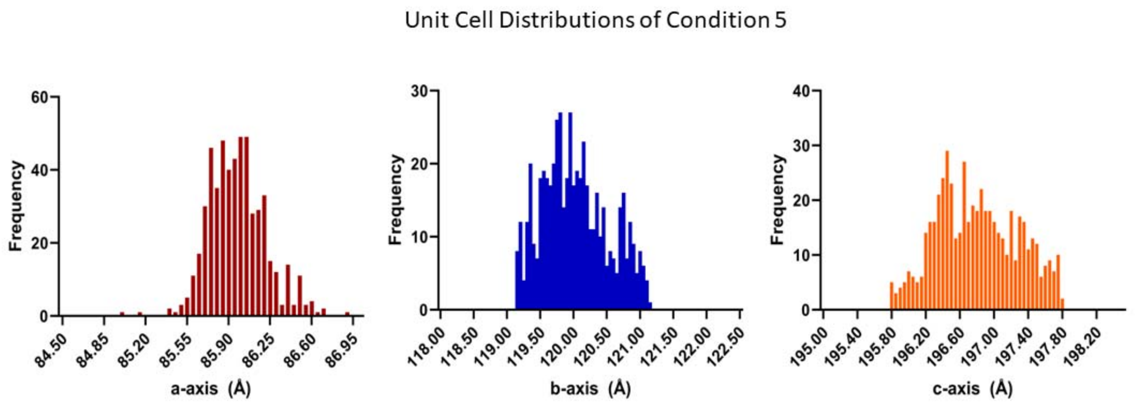

Figure S4 Enlarged unit cell graphs of DtpB screening condition 5. Note that all angles are 90 °.

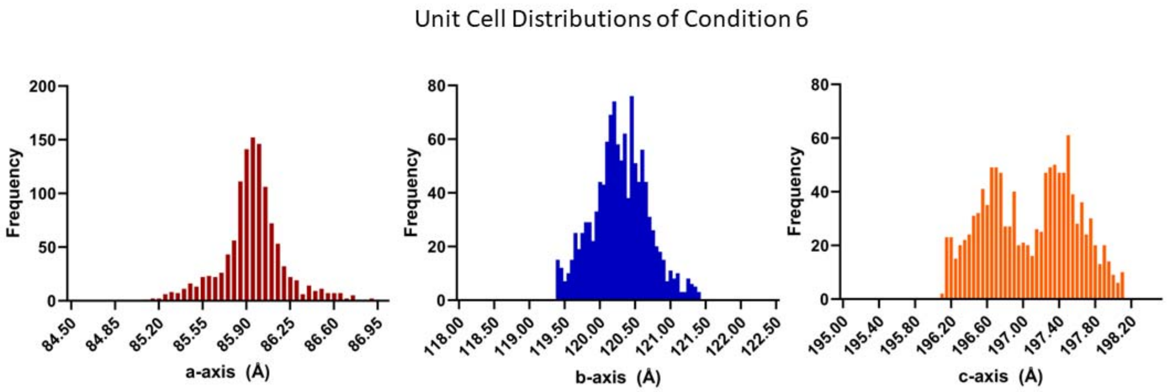

Figure S5 Enlarged unit cell graphs of DtpB screening condition 6. Note that all angles are 90 °.

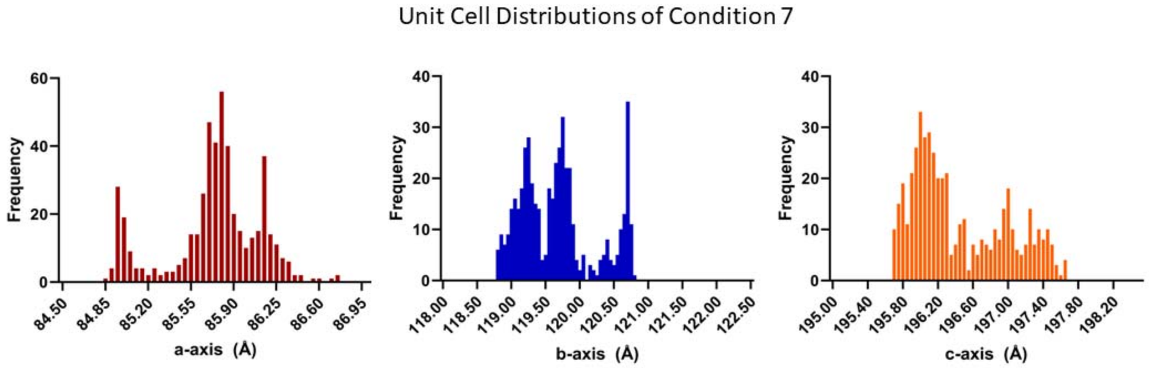

Figure S6 Enlarged unit cell graphs of DtpB screening condition 7. Note that all angles are 90 °.

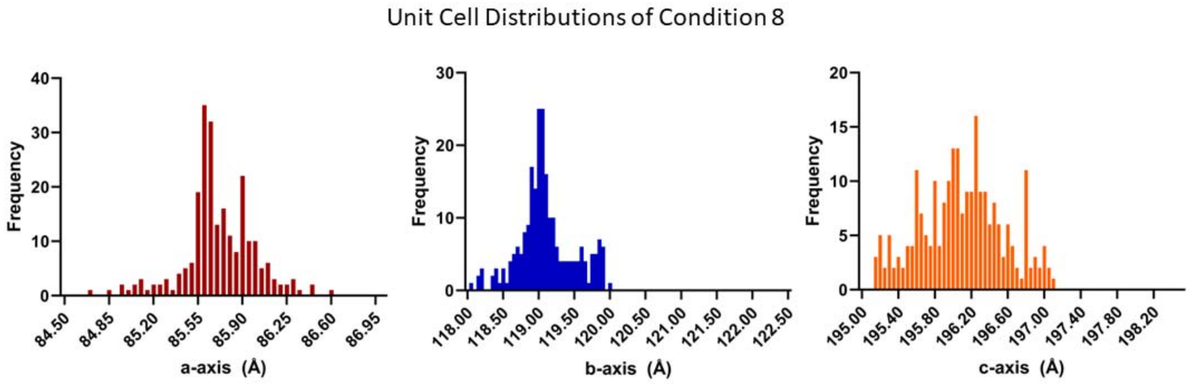

Figure S7 Enlarged unit cell graphs of DtpB screening condition 8. Note that all angles are 90 °.

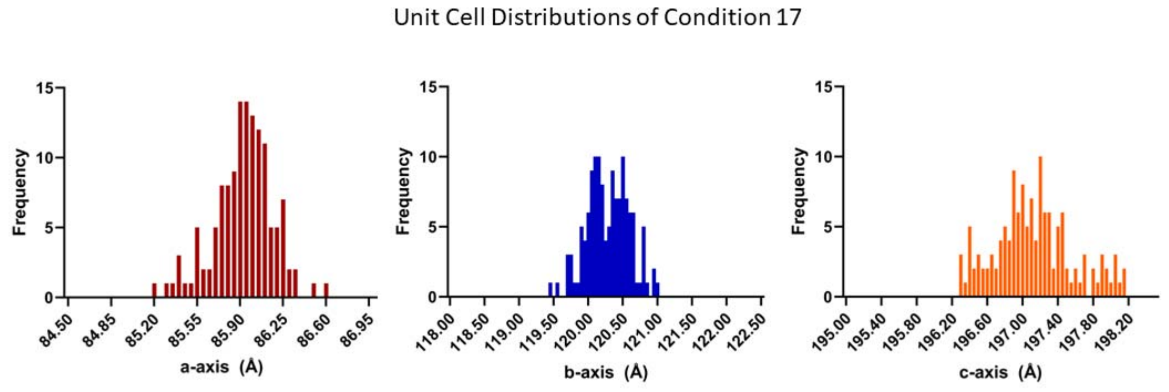

Figure S8 Enlarged unit cell graphs of DtpB screening condition 17. Note that all angles are 90 °.

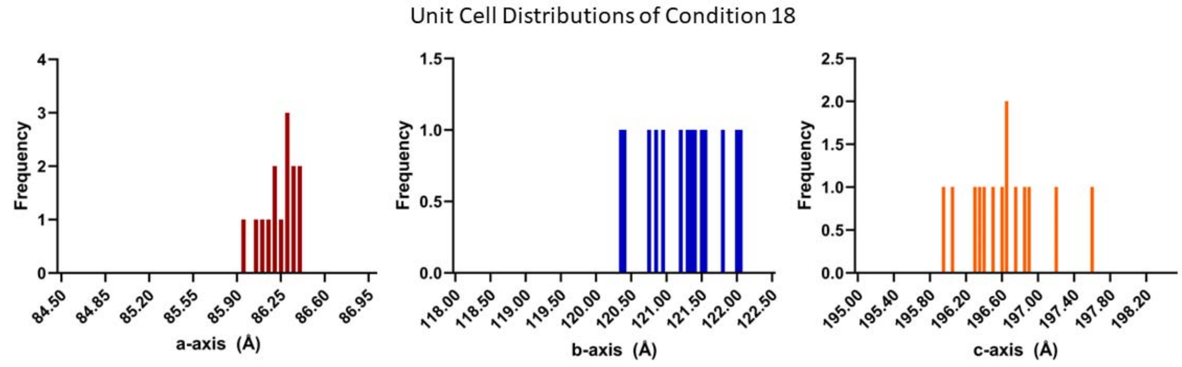

Figure S9 Enlarged unit cell graphs of DtpB screening condition 18. Note that all angles are 90 °.

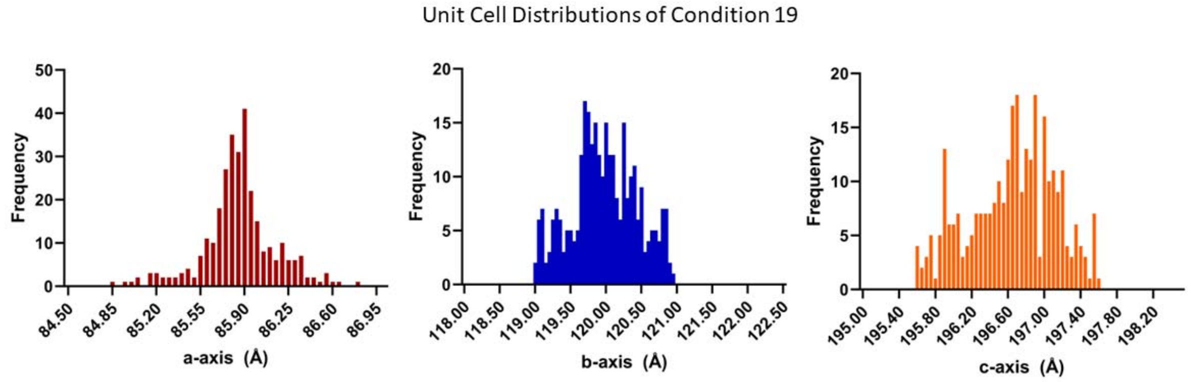

Figure S10 Enlarged unit cell graphs of DtpB screening condition 19. Note that all angles are 90 °.

S5. Comparison of Crystal Quality and Density

As crystal density is a determining factor on how many drops are required for a complete dataset, Figure S11 provides a side-by-side comparison of the samples measured in this study.

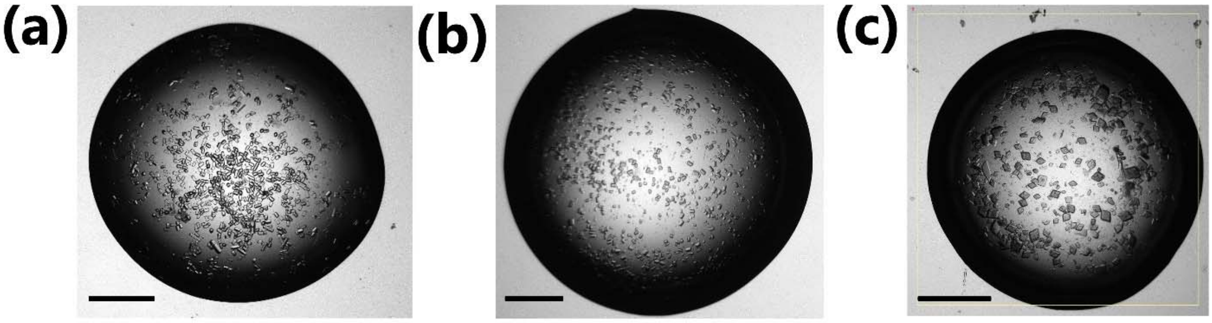

Figure S11 Side-by-side comparisons of crystal drops (a) HEWL (200 nL), (b) DtpAa (100 nL), (c) DtpB (100 nL, condition 18). Heat maps not shown due to significant crystal movement after imaging and prior data collection as plates were moved to the VMXi beamline. All black bars are 200 µm for scale.

S6. RADDOSE Input Files

S6.1. HEWL

```
##################################################
#                     Crystal Block              #
##################################################


Crystal
#hewl


Type Cuboid
Dimensions 25 10 5
PixelsPerMicron 1
AbsCoefCalc  RD3D
UnitCell   78.57 78.57 37.77 90 90 90
NumMonomers  8
NumResidues  129
```

ProteinHeavyAtoms Na 1 Cl 1 S 10

SolventHeavyConc Na 1721 Cl 1711

SolventFraction 0.40

##

Beam Block

##

Beam

Type Gaussian

Flux 2e13

FWHM 10 10

Energy 16

Collimation Rectangular 100 100

###

Wedge Block

###

Wedge 0 0

ExposureTime 0.002

S6.2. DtpAa

##

Crystal Block

##

Crystal

#dtpaa

```
Type Cuboid

Dimensions 30 30 10

PixelsPerMicron 0.5

AbsCoefCalc  RD3D

UnitCell   72.46 67.76 74.91 90 105.69 90

NumMonomers  8

NumResidues  362

ProteinHeavyAtoms Fe 1 S 5

SolventHeavyConc S 75 P 5 Na 120 Cl 38

SolventFraction 0.45

##############################################

#                    Beam Block                    #

##############################################


Beam


Type Gaussian

Flux 2e13

FWHM 10 10

Energy 16

Collimation Rectangular 100 100

###############################################

#                    Wedge Block                    #

###############################################


Wedge 0 0

ExposureTime 0.002
```
